# Improved survival, disparate outcomes contemporaneously define the modern worldwide burden of childhood cancers

**DOI:** 10.3322/caac.70073

**Published:** 2026-02-24

**Authors:** Matthew R. Kudek, Hao Wang

**Affiliations:** ^1^ Medical College of Wisconsin Cancer Center and Department of Pediatrics Medical College of Wisconsin Milwaukee Wisconsin USA; ^2^ Division of Quantitative Sciences, Department of Oncology Johns Hopkins University School of Medicine Baltimore Maryland USA

Over the last 2 decades, patients with childhood cancer collectively have realized significant increases in cure and survival, despite largely stable disease prevalence over the same period. This suggests that an ongoing refinement of therapies, including cellular therapies,^1^ immunotherapies,^2^ and molecularly targeted precision medical therapies,^3^, ^4^ and their incorporation into childhood cancer treatment regimens has improved these outcomes. Indeed, the achievement in mortality reduction enables patients, families, and their medical care teams to replace the devastating news of a childhood cancer diagnosis with the hope for a cure or, perhaps at worst, a manageable chronic disease.

In this issue of *CA: A Cancer Journal for Clinicians*, Li and colleagues^5^ describe recent trends of global childhood cancer statistics over the past 2 decades based on data from the GBD (Global Burden of Disease) 2021 project (source: healthdata.org) and the GLOBOCAN (Global Cancer Observatory) 2022 project (source: International Agency for Research on Cancer). Both the age‐standardized incidence rate and the age‐standardized prevalence rate of childhood cancer declined between 2000 and 2002, rose between 2003 and 2018, then dropped again between 2019 and 2021 during the coronavirus disease 2019 (COVID‐19) pandemic. In contrast, there was a reduction in the age‐standardized mortality rate throughout the entire period. In 2022, there were an estimated 202,164 new childhood cancer cases and 77,182 deaths worldwide. Leukemia remained the most common and fatal childhood malignancy, with brain and central nervous system tumors and non‐Hodgkin lymphoma also contributing substantially to disease burden.

Importantly, the study by Li et al. highlights stark disparities by socioeconomic development. Wealthier nations with higher Human Development Index (HDI) demonstrated higher incidence rates (age‐standardized incidence rate, 15.3 vs. 8.0 per 100,000 in very‐high HDI vs. low HDI regions), likely reflecting better diagnostic infrastructure, whereas poor regions with low HDI experienced disproportionately higher mortality (age‐standardized mortality rate, 4.4 vs. 2.8 per 100,000). Children in low HDI settings face mortality rates greater than 50% higher than their counterparts in very‐high HDI countries, underscoring critical gaps in access to timely diagnosis and effective treatment.

Projections to 2050 reveal a concerning and widening disparity: although medium, high, and very‐high HDI regions are expected to see declines in both new diagnoses and deaths, low HDI countries are projected to experience increases of approximately 45% in both metrics. This trajectory risks deepening the already substantial global disparity in childhood cancer outcomes. It should be noted that the projections from 2022 to 2050 assume stable incidence and mortality rates over time and do not incorporate potential future changes in diagnostic capabilities, treatment advances, or health care infrastructure.

Here, we examine the findings of this study by Li et al. in the context of the COVID‐19 pandemic, population‐based factors associated with mortality trends, and limitations of international disease classifications in global cancer registries for pediatric patients.

## COVID‐19 and childhood cancer trends

When analyzing nearly any modern data set, one must consider how SARS‐CoV‐2 (severe acute respiratory syndrome coronavirus 2) and the associated global COVID‐19 pandemic have influenced the observed data trends. Recognizing the potential for both short‐term and long‐lasting effects of the upheaval caused by the COVID‐19 pandemic spanning early 2020 through mid‐2023,^6^ many have begun to examine the impact of lockdown, stay‐at‐home orders, and quarantines on other public health measures and community health outcomes. For instance, the pandemic resulted in sharp declines in cancer screenings for many of the most common adult cancers,^7^ the effect of which is likely not yet fully realized. In contrast, childhood cancers are not routinely detected by screening methods, so this is not a useful metric to identify trends in clinical access. Instead, one can quantify the effect of the COVID‐19 pandemic on access to care for childhood cancer by examining the number of new cancer cases over a given time period. Indeed, in a comparison of adjusted incidence rates of childhood cancer diagnoses in the United States during 2020 and 2021, decreases of 5% and 4%, respectively, were observed compared with the pre‐pandemic year 2019.^8^ Critically, this likely reflects a delay in diagnosis rather than true decreases in disease incidence. Future studies will be needed to confirm this assumption and evaluate the long‐term effect of these occurrences.

## Population‐based factors associated with childhood cancer mortality

In the data presented by Li and colleagues, a clear dichotomy in survival outcomes was observed: although global mortality of childhood cancer has steadily declined for the last quarter century, normalized mortality rates continue to climb in countries with low HDI scores. Through linear regression analysis, the authors examined population‐based factors associated with significant increases in the mortality‐to‐incidence ratio (MIR) of childhood cancers. Two variables significantly associated with changes in MIR were identified in multivariate analysis. Further study of these factors associated with higher per capita childhood cancer mortality rates is warranted:

(1) Gender Inequality Index (GII): The GII is defined by the United Nations as a composite metric using reproductive health, empowerment, and labor market dimensions. A higher GII, which indicates greater inequality between women and men, was associated with a higher MIR in patients with pediatric cancer. It is important to distinguish here that the MIR is an aggregate of both male and female pediatric patients, suggesting that the *maternal inequalities* experienced influence their *offspring's survival* after the child's childhood cancer diagnosis. One would expect that this higher MIR still portends worse survival for males with childhood cancer, as previously established in other population‐based studies not stratified by GII scores.^9^, ^10^ To address gender disparities in health outcomes for women's cancers, approaches incorporating traditional healers who provide education on the importance of screening and prevention have significantly improved health outcomes.^11^ Similar approaches may also have favorable effects on lowering the MIR, although the interventions need to be carefully crafted for the target population, reflecting both social and cultural sensitivities as well as the contemporary gender norms of the community. Furthermore, the educational messages will need to be tailored to promote the recognition of signs and symptoms of childhood cancers, such as persistent fatigue, newly developed petechiae or easy bruising, prolonged episodes of bleeding, or progressive lymphadenopathy, rather than advocating for screening and prevention measures.
(2) Domestic general government health expenditure (GGHE‐D) as a percentage of current health expenditure (CHE): This is defined by the percentage of health expenditures funded by domestic government sources. In calendar year 2023, published values ranged from 1.7% (Afghanistan) to 92.5% (Brunei Darussalam), as reported by the World Health Organization, with a mean of 52.5%. (source: World Health Organization). In countries with a low GGHE‐D percentage of CHE, larger proportions of health care expenditures are funded out‐of‐pocket. Coupled with low per capita income, as often occurs in impoverished developing regions, families of children who have received a cancer diagnosis are forced to choose between prioritizing daily survival expenses and treatment‐related expenses, creating major financial and psychological strain. Studies have identified economic growth, likely through increases in tax revenue, as a primary driver of increased GGHE‐D in nonhigh‐income countries. This contrasts with high‐income countries, in which changes in public spending, likely driven by policy reform, are a primary driver of increased GGHE‐D.^12^ Consequently, efforts to improve economic conditions may more positively influence childhood cancer outcomes in countries with a low GGHE‐D percentage of CHE than will efforts in policy reform reallocating limited public health funding.

## Constraints of international disease classifications in global cancer registries

Finally, it is important to recognize a current limitation of how global cancer registries limit more accurate childhood cancer classifications. In GLOBOCAN 2022 data, one in four childhood cancer diagnoses remain unspecified by the current international coding strategies for cancers (International Classification of Diseases for Oncology, third edition) because of an anatomic site‐based classification, rather than disease‐specific characteristics. Although the current systems provide a reasonable overview of the anatomic location of a cancer, this approach to characterization is becoming antiquated. Continued advancements in molecular‐based diagnostics require implementation of new classification strategies to accommodate current diagnostic approaches. Other systems, such as those used by the World Health Organization, have adopted strategies to recategorize previously unclassifiable pediatric tumor types on the basis of recurrent molecular aberrations.^13^ To best prioritize scarce resources desperately needed to improve disparate outcomes in childhood cancer survival, it is imperative for major international cancer registries that incorporate childhood cancers to adopt similar strategies.

We close this editorial with optimism for future patients with childhood cancer across the globe. It is important to celebrate improvements in diagnostic processes and multimodal therapeutic advancements, substantiated by annual reductions in the age‐standardized mortality rate worldwide over the last 2 decades. That said, this remains a work in progress, especially for those from impoverished regions of the world who have not yet realized the same increases in survival outlook. As these gaps narrow, there may be new sources of hope among patients with pediatric cancer and their families at a time when they may desperately need it.

## CONFLICT OF INTEREST STATEMENT

The authors declared no conflicts of interest.
